# Riding the risk: A cross-sectional analysis of cycling injuries in the presence of vehicular traffic

**DOI:** 10.1097/MD.0000000000044682

**Published:** 2025-09-26

**Authors:** Namita Bhardwaj, Lewis Tsai, Amy B. Jones, Jayshree S. Thomas, Karen E. Schlag, Wei-Chen Lee

**Affiliations:** a Department of Family Medicine, University of Texas Medical Branch, Galveston, TX; b Department of Orthopaedic Surgery, University of Texas Medical Branch, Galveston, TX; c Sealy Center on Aging, University of Texas Medical Branch, Galveston, TX.

**Keywords:** cycling, injury, sports-related injuries, traffic-related injury

## Abstract

Over the past decade, there has been a surge in the number of individuals participating in cycling. This trend is driven by factors such as increasing awareness of health benefits, environmental concerns, and the promotion of cycling as a sustainable mode of transportation. As cycling continues to grow in popularity, so does the number of cycling-related injuries, suggesting that urban infrastructure may be insufficient to accommodate the rising number of cyclists sharing the road with motor vehicles. This prompts the question: is there a correlation between the prevalence, pattern, and risk factors associated with cycling injuries in the proximity of motor vehicles?. This is a retrospective cohort study using the National Electronic Injury Surveillance System- All Injury Program (NEISS-AIP) to examine cycling injuries between 2006 to 2021. This study compares overall injuries and typical fracture locations in traffic and non-traffic settings. A collective total of 7,032,974 estimated patients were analyzed after incurring cycling-related injuries. Descriptive analytics and bivariate analyses were used to characterize the data. Results revealed more cycling injuries in the presence of vehicular traffic than non-traffic (weighted 44.1% > 25.1%) and the most common injuries were to the head and face (19.9% and 14.0%, *P* < .001), and included injuries such as facial lacerations wrist fractures and knee contusions. Males were injured more than females (73.5% and 26.5% *P* < .0002). Most injuries were treated in the emergency department (90.5%, *P*-value <.001). Overall, data analysis showed that there was a statistically significant increase in the number of injuries in traffic-related conditions as opposed to those in non-traffic settings, yet no significant difference in the type of injuries in both groups. This study offers insights that may potentially shape preventative measures including equipping hospital medical clincians on the treatment of most common diagnoses, directing first responder training during mass participation events, and informing public policy makers on safety initiatives in regions with a high prevalence of cycling and presence of traffic.

## 1. Introduction

Cycling is popular both as a recreational activity and a method of transportation. Approximately 32% of Americans rode a bicycle recreationally in 2018.^[1]^ The increasing popularity of cycling in recent decades has resulted in an increased number of crashes as cyclists and automobiles struggle to share the road.^[2]^ “According to the National Highway Traffic Safety Administration, 1105 bicyclists were killed in motor-vehicle traffic crashes in 2022 (latest data available), a 13% increase from 976 in 2021.”^[3]^ Cyclists involved in a crash are at significant risk for injuries such as blunt injuries and fractures.^[4]^ Yet less is known about whether the pattern of injuries sustained in a cycling crash changes based on whether a vehicle was involved. Previous studies looking at mountain biking injuries show a large number of injuries impacting the head and neck.^[5]^ Foley et al evaluated injuries impacting cyclists in Ireland and found that only 30% of cases are related to a motor vehicle.^[6]^ The other 70% of injuries were related to unspecified, obstacles, fellow cyclists or mountain biking.^[6]^ They found that most people sustained head injuries.^[6]^ Limited literature exists on the impact of cycling injuries presenting to the emergency departments in the United States and the relationship with motor vehicles.

This paper examines the relationship between the presence or absence of motor vehicles and the number of injuries sustained by a cyclist as well as the specific type(s) of injury in each of the 2 circumstances. An improved understanding of this relationship has important implications for 3 primary groups of stakeholders. Municipalities’ planning and transportation departments that must plan roadways as there are arguments for and against dedicated bike lanes or streets.^[2]^ Initial work by Reynolds showed that having well thought out bike lanes reduced injuries,^[7]^ but Wee et al showed that while more people are utilizing bike lanes, they are not safer compared to non-bike lane injuries^[8]^; As the interest in triathlons has grown,^[9]^ planners and coordinators of cycling events who must decide whether to share the road with vehicles^[4]^ during the event or spend a much larger amount of money and resources to completely close down a road to vehicular traffic.^[5]^ Limited resources on the potential impact of open or closed roads during events exist in the literature; first responders and medical personnel who must prepare for specific injuries they may see when responding to a vehicle or non-vehicle-involved accident and decide what equipment and personnel to have readily available on site and at treatment facilities, such as emergency departments.”

## 2. Methods

### 2.1. Data source

This is a secondary analysis of data from the United States Consumer Product Safety Commission’s National Electronic Injury Surveillance System – All Injury Program database (NEISS-AIP) for the years 2006 to 2021. NEISS-AIP collects data on consumer product-related injuries occurring in the United States and offers weight variables to produce nationwide estimate of injuries.^[10–25]^ NEISS-AIP also collects patient information from a nationally representative sample of hospital emergency departments such as diagnosis, body part, and drug-involved accident. We included all injuries with “pedal cyclists” as the cause for analysis. Specifically, this would mean injury to a pedal cycle rider resulting from a collision, loss of control, crash, or some other event involving a moving vehicle or pedestrian.^[10–25]^ Next, we classified all injuries into 3 incident types: traffic related, non-traffic related, or unknown. Traffic-related injuries included those where a vehicle was involved on a public road. Non-traffic related injury included any incident that occurred entirely in any place other than a public highway, street, or road. Unknown injury could be any incident that was did not have a report specifying whether the incident happened on a public highway, street, or road.

### 2.2. Data analysis

The primary outcome was the total frequency of injuries for each of the 3 incident types for each year between 2006 and 2021. Next, for each incident type, the study examined patient characteristics including gender, age, race, injury location (e.g. head, face), diagnosis (e.g. contusion, fracture), and disposition (e.g. treated, hospitalized). Finally, the study examined the cross tabulations between top 3 diagnoses (contusion, fracture, and laceration) and eleven injury body locations by 3 incident types. We applied the weight variables generated by NEISS-AIP to reflect the national estimates for injury prevalence including lower control limits (LCL) and upper control limits (UCL). We removed missing data from the analysis to minimize bias.

We performed descriptive analytics of the total frequencies by incident type and by year to demonstrate trends in pedal cycling-related injuries using one-Way ANOVA. Next, we conducted analyses to examine the patient characteristics and injury characteristics for each incident type with Chi-Squared tests. Finally, we performed another cross tabulation to compare the most prevalent injury body location for each of the top 3 diagnoses and for each of the 3 incident types. We considered any *P*-value <.05 to be statistically significant. All analyses were done using STATA v18 (College Station, TX).

The University of Texas Medical Branch Institutional Review Board (FWA#: 00002729) reviewed our study and declared it to be exempt from review by the IRB as this is secondary data analysis for which consent is not required.

## 3. Results

### 3.1. Injury total

Overall, the total injury frequency from the years 2006 to 2021 changed ranging from a low of 22,887,137 in 2020 to a high of 32,074,270 in 2016. The trend analysis was statistically significant (*P* = .0055) in which the unknown frequencies increased while traffic related frequencies decreased. Most injuries often happened when there was traffic (3,103,235), compared to non-traffic (1,767,109) and unknown presence of vehicles (2,163,594). When evaluating injury demographics, males (73.5%) were more likely to be impacted than females (26.5%) based on Table 1. Those with injuries in traffic related conditions had an average age of 32.9 years, compared to those with non-traffic related injuries at 21.3 years or unknown with 24.25 years (*P*-value <.001).

**Table 1:** Injury prevalence.

	Freq.	Wgt Freq.	LCL Wgt Freq.	UCL Wgt Freq.	Wgt Col %	LCL Wgt Col %	UCL Wgt Col %
Gender (missing = 8)							
Male	95,270	5,170,151	4,027,175	6,313,127	73.5	71.8	75.1
Female	33,754	1,862,823	1,412,595	2,313,052	26.5	24.9	28.2
Top body types (no missing)							
Head	22,104	1143,130	790,382	1,495,877	16.3	14.5	17.3
Face	14,760	802,136	630,408	973,863	11.4	11.6	11.3
Shoulder	9816	605,081	445,351	764,811	8.6	8.2	8.9
Knee	9172	544,308	432,885	655,731	7.7	8.0	7.6
Lower trunk	7082	404,707	287,756	521,658	5.8	5.3	6.0
Upper trunk	6458	387,387	281,404	493,369	5.5	5.2	5.7
Lower leg	7044	363,486	280,416	446,555	5.2	5.2	5.2
Elbow	6881	387,873	308,664	469,083	5.5	5.7	5.4
Wrist	7731	455,088	359,043	531,133	6.3	6.6	6.2
Lower arm	7777	360,315	283,444	437,186	5.1	5.2	5.1
Ankle	5395	300,510	239,529	361,490	4.3	4.4	4.2
Top diagnoses (no missing)							
Contusion	32,738	1,879,660	1,494,113	2,265,207	26.7	27.5	32.2
Fracture	31,511	1,660,559	1,212,284	2,108,834	23.6	24.7	28.0
Laceration	22,538	1,233,932	984,857	1,483,007	17.5	18.3	20.8
Internal injury	14,063	724,309	452,498	996,119	10.3	9.2	14.2
Strain/sprain	13,543	808,406	649,480	967,331	11.5	11.8	14.0
Disposition (missing = 9)							
Treated	114,946	6,363,071	4,995,884	7,730,258	90.5	88.0	92.5
Hospitalized	10,750	471,204	238,400	704,008	6.7	4.7	9.5
Transferred	1069	90,703	66,493	114,914	1.3	0.9	1.7
AMA/LWBS	1396	73,898	43,167	104,628	1.1	0.7	1.5
Observation	862	33,994	13,595	54,393	0.5	0.3	0.8

AMA = a patient leaves against medical advice, Col = Column, Freq = frequency, LCL = lower control limit, LWBS = leaves without being seen, UCL = upper control limit, Wgt = weighted.

**Figure 1.**
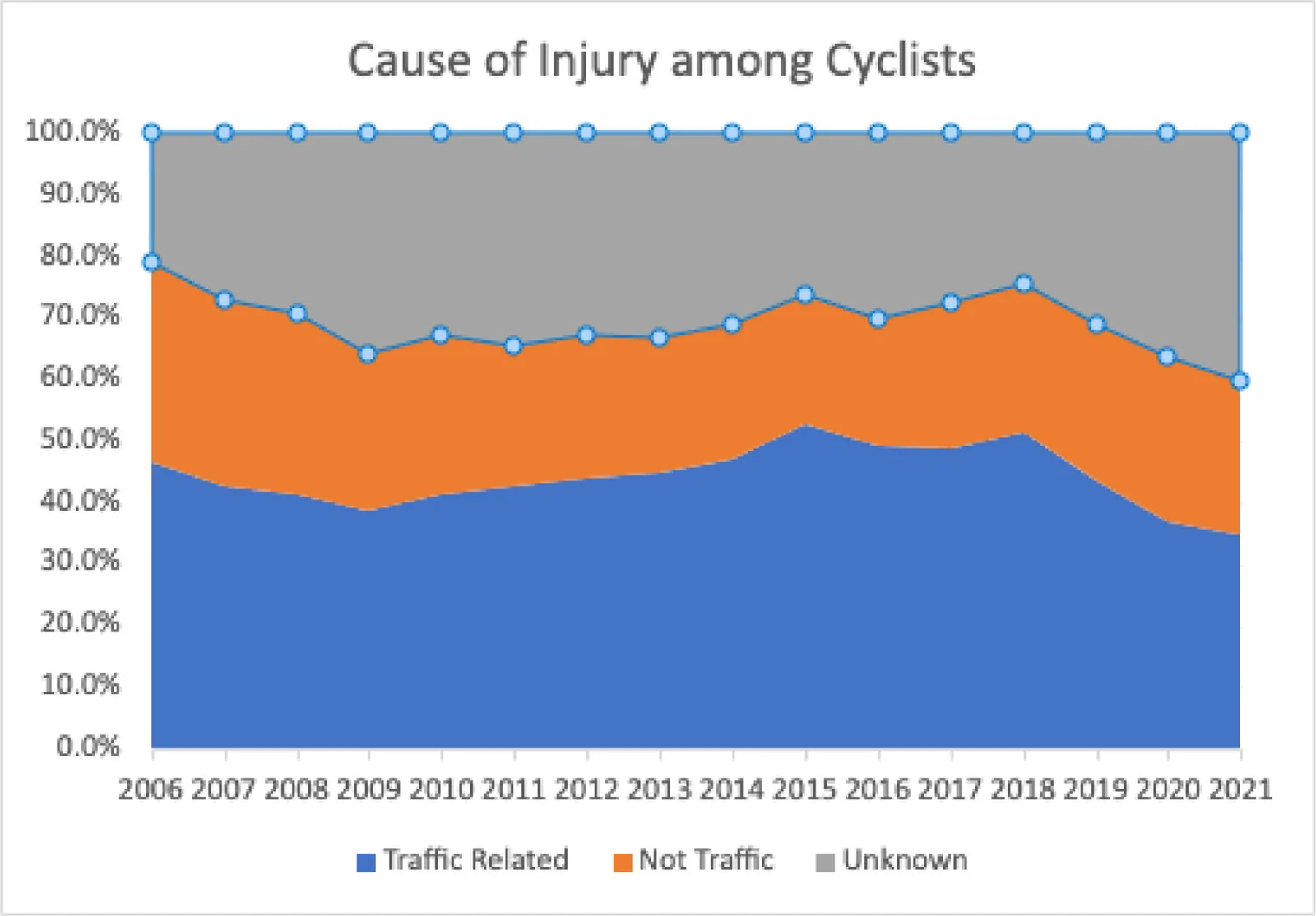
Cause of Injury among cyclists.

Figure 1 depicts injury totals by incident type and by year, and Table 1 depicts demographic trends.

### 3.2. Injury body location

The average age was 27.4 years based on Table 2. Interestingly, those in traffic related injuries had a higher age of 32.9 years and compared to those not in traffic of 21.3 years (*P* < .001). Most often whites (74.0%) were impacted by the injuries compared to other races/ethnicities but not significantly (*P* = .5268). When comparing the body part impacted by the cycling injury, injuries to the head, face, or shoulder more likely regardless of incident type (*P* < .001). Also, the diagnosis and disposition were significantly different when comparing vehicular and non-vehicular settings (*P* = .003 and *P* < .0001). Traffic-related injuries resulted in contusion more than non-traffic-related injuries (44.0% > 35.5%). Next, traffic-related injuries were more frequently resulted in hospitalization than non-traffic-related injuries (9.8% > 4.1%).

**Table 2:** Injury demographics.

**Weighted N (%**)	**Total**	**Traffic**	**Not traffic**	**Unknown**	***P*-value**
Unweighted N = 1,29,032					
Gender (missing = 8)					.0002
Male (n, %)	5,170,151 (73.5%)	2,359,442 (76.0%)	1,255,079 (71.0%)	1,555,630 (71.9%)	
Female (n, %)	1,862,823 (26.5%)	743,781 (24.0%)	511,940 (29.0%)	607,103 (28.1%)	
Age (missing = 24)	27.4 (1.33)	32.9 (1.19)	21.3 (1.01)	24.5 (1.26)	<.001
Race & ethnicity (missing = 32,188)					.5268
White	3,974,433 (74.0%)	1,729,906 (72.0%)	1,118,751 (76.8%)	1125,775 (74.4%)	
Black	675,346 (12.6%)	319,850 (13.3%)	161,438 (11.1%)	194,059 (12.8%)	
Hispanic	534,031 (9.9%)	262,750 (10.9%)	124,312 (8.5%)	146,969 (9.7%)	
Asian	104,800 (2.0%)	56,383 (2.4%)	25,306 (1.7%)	23,112 (1.5%)	
American Indian	38,666 (0.7%)	15,955 (0.7%)	15,744 (1.1%)	6967 (0.5%)	
Others	45,342 (8.4%)	17,710 (0.7%)	11,742 (0.8%)	15,889 (1.1%)	
Body location (no missing)					<.001
Head	1,143,130 (16.3%)	568,656 (18.3%)	270,330 (15.3%)	304,143 (14.1%)	
Face	802,136 (11.4%)	319,918 (10.3%)	251,216 (14.2%)	231,002 (10.7%)	
Shoulder	605,081 (8.6%)	304,403 (9.8%)	133,471 (7.6%)	167,206 (7.7%)	
Knee	544,308 (7.7%)	252,511 (8.1%)	125,393 (7.1%)	166,403 (7.7%)	
Lower trunk	404,707 (5.8%)	220,216 (7.1%)	71,210 (4.0%)	113,281 (5.2%)	
Upper trunk	387,387 (5.5%)	203,875 (6.6%)	77,751 (4.4%)	105,760 (4.9%)	
Lower leg	363,486 (5.2%)	166,025 (5.4%)	83,596 (4.7%)	113,865 (5.3%)	
Elbow	387,873 (5.5%)	165,413 (5.3%)	100,682 (5.7%)	122,778 (5.7%)	
Wrist	455,088 (6.3%)	162,825 (5.3%)	123,351 (7.0%)	158,912 (7.4%)	
Lower arm	360,315 (5.1%)	128,255 (4.1%)	98,343 (5.6%)	133,716 (6.2%)	
Ankle	300,510 (4.3%)	119,878 (3.9%)	76,562 (4.3%)	104,070 (4.8%)	
Diagnosis (no missing)					.0003
Contusion	1,879,660 (26.7%)	927,906 (29.9%)	446,669 (25.3%)	505,085 (23.4%)	
Fracture	1,660,559 (23.6%)	750,824 (24.2%)	403,718 (22.8%)	506,019 (23.4%)	
Laceration	1,233,932 (17.5%)	431,430 (13.9%)	406,884 (23.0%)	395,618 (18.3%)	
Internal injury	724,309 (10.3%)	372,750 (12.0%)	151,750 (8.6%)	199,809 (9.2%)	
Strain/sprain	808,406 (11.5%)	346,754 (11.2%)	191,686 (10.8%)	269,966 (12.5%)	
Disposition (missing = 9)					<.001
Treated	6,363,071 (90.5%)	2,709,177 (87.3%)	1,652,198 (93.5%)	2,001,696 (92.5%)	
Hospitalized	471,204 (6.7%)	304,626 (9.8%)	72,316 (4.1%)	94,262 (4.4%)	
Transferred	90,703 (1.3%)	36,618 (1.2%)	26,956 (1.5%)	27,129 (1.3%)	
AMA/LWBS	73,898 (1.1%)	33,888 (1.1%)	8961 (0.5%)	31,048 (1.4%)	
Observation	33,994 (0.5%)	18,834 (0.6%)	6654 (0.4%)	8505 (0.4%)	

A patient leaves against medical advice (AMA) or leaves without being seen (LWBS).

### 3.3. Disposition

When evaluating disposition in Table 2, for all incident types combined, injuries most often resulted in treatment in the emergency department (90.5%), hospitalization (6.7%), transfer to another facility (1.3%), the patient leaving against medical advice (AMA) or leaving without being seen (LWBS) (1.1%), and observation (0.5%). The incident type did have a significant impact on disposition (*P* < .001). This shows that most injuries were not significant enough to warrant a hospitalization, but were able to be addressed by the emergency department.

### 3.4. Diagnoses

The most common diagnoses in a combined analysis of both traffic and non-traffic injuries as shown in Table 3, were facial lacerations (49.7% of all laceration cases), knee contusions (17.3% of all laceration cases), facial contusions (14.3% of all contusion cases), shoulder (18.6%) and wrist fractures (17.3% of all fracture cases), and lower leg lacerations (12.9 of all laceration cases). The only difference is that for both non-traffic (19.7%) or unknown injuries (22.6%), lower arm is the most commonly fractured body part but for traffic-related injuries, shoulder (19.8%) is the most commonly fractured body part.

**Table 3:** Body part impacted by diagnosis.

Body type	Contusion	Fracture	Laceration
Weighted N = 3,831,197 (mean and standard error)	Top 3 diagnoses (despite the cause)		
Head	91,396 (5.9 ± 0.8%)	19,337 (1.4 ± 0.4%)	128,366 (14.0 ± 1.2%)
Face	222,876 (14.3 ± 1.1%)	99,061 (7.3 ± 0.8%)	455,972 (49.7 ± 2.4%)
Shoulder	150,123 (9.7 ± 0.7%)	252,593 (18.6 ± 1.2%)	2246 (0.2 ± 0.09%)
Knee	267,975 (17.3 ± 0.8%)	29,066 (2.1 ± 0.3%)	99,017 (10.8 ± 1.1%)
Lower trunk	188,947 (12.2 ± 1.3%)	86,737 (6.4 ± 1.3%)	12,849 (1.4 ± 0.3%)
Upper trunk	194,989 (12.6 ± 1.1%)	121,241 (8.9 ± 1.2%)	4445 (0.5 ± 0.1%)
Lower leg	115,011 (7.4 ± 0.7%)	90,049 (6.6 ± 0.5%)	118,669 (12.9 ± 1.2%)
Elbow	171,234 (11.0 ± 0.7%)	120,845 (8.9 ± 0.7%)	42,930 (4.7 ± 0.6%)
Wrist	38,075 (2.5 ± 0.4%)	234,778 (17.3 ± 1.9%)	4073 (0.4 ± 0.1%)
Lower arm	75,755 (4.9 ± 0.7%)	232,527 (17.1 ± 2.6%)	30,608 (3.3 ± 0.5%)
Ankle	37,034 (2.4 ± 0.3%)	73,621 (5.4 ± 0.6%)	18,750 (2.0 ± 0.2%)
Total	1,553,415 (100%)	1,359,856 (100%)	917,925 (100%)
Weighted N = 1,747,272	Top 3 diagnoses (traffic related)		
Head	41,645 (5.3 ± 0.8%)	12,947 (2.1 ± 0.7%)	52,194 (15.5 ± 1.7%)
Face	95,137 (12.1 ± 1.4%)	51,947 (8.4 ± 1.3%)	164,371 (48.8 ± 3.5%)
Shoulder	85,236 (10.8 ± 0.9%)	123,396 (19.8 ± 1.8%)	1327 (0.4 ± 0.8%)
Knee	143,101 (18.1 ± 0.7%)	16,237 (2.6 ± 0.4%)	33,856 (10.1 ± 1.2%)
Lower trunk	105,132 (13.3 ± 2.0%)	54,364 (8.7 ± 1.5%)	5020 (1.5 ± 0.5%)
Upper trunk	101,832 (12.9 ± 1.1%)	70,751 (11.4 ± 1.5%)	1545 (0.5 ± 0.1%)
Lower leg	67,327 (8.5 ± 1.0%)	46,299 (7.4 ± 0.6%)	37,851 (11.2 ± 1.7%)
Elbow	82,393 (10.4 ± 0.7%)	47,967 (7.7 ± 0.8%)	19,230 (5.7 ± 0.8%)
Wrist	15,394 (2.0 ± 0.5%)	90,169 (14.5 ± 1.9%)	1783 (0.5 ± 0.2%)
Lower arm	34,955 (4.4 ± 0.9%)	75,500 (12.1 ± 2.2%)	12,555 (3.7 ± 0.9%)
Ankle	16,471 (2.1 ± 0.4%)	32,567 (5.2 ± 1.0%)	6773 (2.0 ± 0.5%)
Total	788,623 (100%)	622,144 (100%)	336,504 (100%)
Weighted N = 984,967	Top 3 diagnoses (non-traffic related)		
Head	29,661 (8.3 ± 1.7%)	3446 (1.0 ± 0.3%)	41,487 (14.0 ± 1.4%)
Face	66,080 (18.4 ± 1.8%)	22,911 (7.0 ± 1.2%)	155,980 (52.7 ± 2.7%)
Shoulder	30,796 (8.6 ± 1.2%)	57,414 (17.4 ± 1.4%)	456 (0.2 ± 0.1%)
Knee	55,817 (15.5 ± 1.6%)	4487 (1.4 ± 3.5%)	33,408 (11.3 ± 1.6%)
Lower trunk	36,535 (10.2 ± 1.2%)	13,203 (4.0 ± 1.07%)	3263 (1.1 ± 0.3%)
Upper trunk	42,109 (11.7 ± 1.3%)	21,553 (6.5 ± 1.0%)	1042 (0.4 ± 0.2%)
Lower leg	20,200 (5.6 ± 0.6%)	20,531 (6.2 ± 0.8%)	34,737 (11.7 ± 1.4%)
Elbow	39,862 (11.1 ± 0.9%)	35,450 (10.7 ± 1.09%)	10,739 (3.6 ± 0.8%)
Wrist	10,267 (2.9 ± 0.6%)	66,529 (20.2 ± 3.3%)	794 (0.3 ± 0.1%)
Lower arm	18,407 (5.1 ± 0.7%)	65,006 (19.7 ± 3.5%)	9005 (3.0 ± 0.6%)
Ankle	9620 (2.7 ± 0.5%)	19,308 (5.9 ± 1.2%)	4864 (1.6 ± 0.4%)
Total	359,354 (100%)	329,838 (100%)	295,775 (100%)
Weighted N = 1,098,958	Top 3 diagnoses (unknown cause of cycling injury)		
Head	20,090 (5.0 ± 0.9%)	2944 (0.7 ± 0.3%)	34,686 (12.1 ± 1.7%)
Face	61,660 (15.2 ± 1.1%)	24,203 (5.9 ± 0.9%)	135,621 (47.5 ± 2.6%)
Shoulder	34,091 (8.4 ± 0.9%)	71,783 (17.6 ± 1.7%)	463 (0.2 ± 0.08%)
Knee	69,056 (17.0 ± 1.1%)	8342 (2.1 ± 0.4%)	31,754 (11.1 ± 1.54%)
Lower trunk	47,280 (11.7 ±.4%)	19,170 (4.7 ± 1.2%)	4565 (1.6 ± 0.4%)
Upper trunk	51,048 (12.6 ± 1.5%)	28,937 (7.1 ± 1.4%)	1858 (0.7 ± 0.2%)
Lower leg	27,485 (6.8 ± 1.0%)	23,220 (5.7 ± 0.7%)	46,081 (16.1 ± 1.7%)
Elbow	48,979 (12.1 ± 1.2%)	37,427 (9.2 ± 1.0%)	12,962 (4.5 ± 0.7%)
Wrist	12,413 (3.1 ± 0.7%)	78,080 (19.1 ± 2.8%)	1496 (0.5 ± 0.2%)
Lower arm	22,392 (5.5 ± 0.9%)	92,021 (22.6 ± 3.6%)	9048 (3.2 ± 0.6%)
Ankle	10,943 (2.7 ± 0.5%)	21,746 (5.3 ± 0.6%)	7112 (2.5 ± 0.6%)
Total	405,438 (100%)	407,874 (100%)	285,646 (100%)

## 4. Discussion

The primary aim of this paper was to evaluate the trends between the presence or absence of motor vehicles on the injuries sustained by cyclists. Our results show that cycling injuries trend more with incidents involving a vehicle. Cyclists that sustain injuries related to motor vehicles had worse outcomes than those not involved with motor vehicles.^[26]^ We found that contusions and lacerations were more common, which contrasts with another study by Rooney et al and evaluated only road cycling injuries which found lacerations and fractures were most common.^[27]^ When evaluating non-traffic injuries, we found that the most common location of injuries were to the head and hand which is consistent with work done by Kshirsagar (2022).^[28,29]^ Since 2006 to 2021, cycling injuries which present to emergency departments have remained relatively stable, but both traffic and non-traffic related cyclist injuries are decreasing.

Cycling is also one of the components of triathlons which include such events as the Iron Man race. These races often involve long distances on roads, including up to 112 miles of cycling during the Iron Man triathlons. When race directors are planning such events, they must obtain permits to not only hold the event, but also to have the ability to have the athletes participate safely.^[30]^ One must consider the costs about stopping vehicular traffic during the course of the event for athlete safety or to permit vehicular traffic to minimize impact to the surrounding community.^[31]^ Based the results of our paper, we show that shoulder injuries are more common in traffic related accidents and lower arm is more common in unknown or non-traffic situations. Increased awareness and knowledge of injury patterns may help emergency personnel be better prepared to address emergencies situations, such as having more slings in place to stabilize shoulder injuries. Knowing that there will be fewer injuries in a completely closed road bike event may spur the organizer to choose that path and therefore use potential funds saved on medical equipment/personnel to pay for more police officers to help close and monitor roads. Professionals have lower injury rates than amateurs but given that most of these racing events involve athletes at all levels, one must be prepared for all injury types.^[27]^ However, money saved on opting for a shared-road event may be used on more medical personnel/stations as you will likely see an increased number of accidents/injuries.

Injury body location and injury type are pertinent information to provide local emergency departments to help them plan for specific specialists to be more readily available during mass participation cycling events such as plastic surgery for complex facial lacerations, and orthopedic hand surgeons for wrist injuries. If roads are open to traffic, orthopedic shoulder specialists may be informed of the possibility of an increased number of consultations. Additionally, medical clinicians during cycling events may consider supplying additional materials to treat facial lacerations or splint upper extremity fractures.

Although we can safely assume the frequency of injuries is greater if a vehicle is involved, the fact that the significance is smaller may not do much to help quell the debates of whether to include dedicated bike lanes. A study by Gu et al showed that “over the lifetime of all people in NYC, bike lane construction produces additional costs of $2.79 and gain of 0.0022 quality-adjusted life years per person.” Contrast that to a study by Wee et al showing that more injuries occur to cyclists in bike lanes compared to non-bike lanes.^[8]^ The information here can be used in the fashion of a factor to identify on a list of pros and cons that engineers and city planners can use to help decide where to dedicate their resources.

### 4.1. Implications for future research

Future directions may involve evaluating economic costs of road closure versus non-road closure for athletic events and how city planners may best utilize this information to inform their decision-making process. There has been an increase in the number of cycling injuries during the 15 years evaluation period and a deeper dive into the reasons behind this increase and how to minimize those injuries may be fruitful for further evaluation.

### 4.2. Limitations

Limitations of the paper include that while the data is national representative sample, one is not able to accurately identify the mechanism of injury nor the context of the actual situation surrounding the injury. We are limited to classifications based on the collectors for the NEISS-AIP database. As minor injuries may not present to the emergency department, we may not be capturing those in our analysis. Several of the injuries were due to unknown causes, most likely due to inadequate documentation in the medical records. A more thorough evaluation of the unknown mechanisms of injuries, would help to better characterize the true trends of the injuries.

## 5. Conclusion

It is the nature of recreational cycling and cycling events to be on the roads with and without vehicles. Based on the analysis of data pulled from NEISS-AIP, there is an expected increase in cycling injuries in traffic versus non traffic roads. There is no significant difference in types of injuries between the 2 groups, but the disposition and diagnoses do differ; however, there is pattern of injuries that can be helpful in determining how local emergency departments and mass participation event medical clinicanspro can prepare. Facial lacerations are by far the most common laceration injury, wrist fractures the most common fracture, and knee contusions the most common contusion injury. Not only is this information pertinent for medical clinicians, but also policy makers when addressing public safety in areas where cycling is more prevalent.

## Author contributions

**Conceptualization:** Namita Bhardwaj, Lewis Tsai, Karen E. Schlag.

**Data curation:** Namita Bhardwaj, Lewis Tsai, Amy B. Jones, Jayshree S. Thomas, Wei-Chen Lee.

**Formal analysis:** Namita Bhardwaj, Lewis Tsai, Karen E. Schlag, Wei-Chen Lee.

**Investigation:** Namita Bhardwaj, Lewis Tsai, Amy B. Jones, Jayshree S. Thomas, Wei-Chen Lee.

**Methodology:** Namita Bhardwaj, Lewis Tsai, Amy B. Jones, Jayshree S. Thomas, Karen E. Schlag, Wei-Chen Lee.

**Project administration:** Namita Bhardwaj, Jayshree S. Thomas, Karen E. Schlag, Wei-Chen Lee.

**Resources:** Namita Bhardwaj.

**Supervision:** Namita Bhardwaj.

**Visualization:** Namita Bhardwaj.

**Writing – original draft:** Namita Bhardwaj, Lewis Tsai, Amy B. Jones, Jayshree S. Thomas, Karen E. Schlag, Wei-Chen Lee.

**Writing – review & editing:** Namita Bhardwaj, Lewis Tsai, Amy B. Jones, Jayshree S. Thomas, Karen E. Schlag, Wei-Chen Lee.
